# Clinical value of artificial intelligence computer-aided diagnosis system in lymph node ultrasonography: A STARD-compliant article

**DOI:** 10.1097/MD.0000000000050853

**Published:** 2026-09-25

**Authors:** Tingting Jin, Liying Wang, Shusen Zheng

**Affiliations:** a Department of Ultrasound, Shaoxing Second Hospital, Shaoxing, Zhejiang, China; b Key Laboratory of Combined Multi-Organ Transplantation, Ministry of Public Health, Zhejiang University, Key Laboratory of Organ Transplantation, Hangzhou, Zhejiang, China.

**Keywords:** artificial intelligence, characteristic parameter, computer-aided diagnosis, lymph node, ultrasonography

## Abstract

This article explores the clinical value of the artificial intelligence computer-aided diagnosis system in the diagnosis of superficial lymph nodes. In this study, characteristic ultrasonic parameters of 185 cases of lymph nodes (including 110 benign and 75 malignant) confirmed by pathology were quantified by the superficial lymphadenopathy computer-aided diagnosis system, including margin, appearance, medulla proportion, medulla distribution, echogenicity, echogeneity, vascular density, and vascular pattern. A nonparametric rank sum test was used between the parameters. The sensitivity, specificity, and accuracy of the artificial intelligence computer-aided diagnosis system in the diagnosis of superficial lymph nodes were 80.0%, 80.9%, and 80.5%. The characteristics of margin, appearance, medulla proportion, medulla distribution, echogenicity, echogeneity, vascular density, and vascular pattern could identify benign and malignant lymph nodes. The artificial intelligence superficial lymphadenopathy computer-aided diagnosis system can screen out the characteristic ultrasonic parameters of benign and malignant lymph nodes, which has important value in lymph node clinical diagnosis.

## 1. Introduction

Artificial intelligence (AI) is a branch of computer science, and its essence is to simulate human intelligence in processing all kinds of information and tasks. Machine learning is the core technology of artificial intelligence in imitating human learning, and through the study and analysis of medical data, it provides effective auxiliary methods for disease diagnosis, treatment, and prognosis prediction. Artificial intelligence computer-aided diagnosis (CAD) refers to the use of image processing technology, such as the feature parameter extraction of certain diseases, analysis, and comparison with the large statistical data of similar examinations in a computer-aided diagnosis system, and making the diagnosis conclusion.^[1]^ With the rapid development of picture archiving and communications systems and their wide applications in clinical fields, the functions of medical image acquisition and transmission, large-capacity data storage, image display, and processing can be realized, which promotes the development of computer-aided diagnosis technology. The initial computer-aided diagnosis research work mainly focused on the pneumoconiosis examination of pneumoconiosis patients. With the development of automation technology, image processing technology, pattern recognition technology, and information technology, as well as the breakthrough of mathematical tools, these technologies gradually infiltrate into medical diagnosis and promote the development of CAD.^[2]^

Lymphadenopathy is a common clinical manifestation. The sensitivity of ultrasound is better than that of computed tomography and magnetic resonance imaging. Therefore, ultrasound is the preferred imaging examination method for lymph node lesions.^[3]^ However, ultrasound examination is interfered with by experts’ technical level and subjective factors. In recent 10 years, the research on computer-aided diagnosis of ultrasound images at home and abroad mainly focused on liver diseases and breast diseases.^[4–6]^Gatos created an SVM model that quantifies color information in terms of stiffness from shear wave elastography images, which distinguished patients with liver diseases from controls with 87.3% accuracy, 93.5% sensitivity, and 81.2% specificity.^[7]^

The professional level and experience of ultrasound doctors often directly determine the accuracy of diagnosis, so the ultrasound diagnosis of lymph nodes is vulnerable to interference from experts’ technical level and doctors’ subjective factors. The essence of computer is thinking simulation and powerful storage capacity, which can store all information of lymph nodes in computer. Secondly, the memory of a computer can be transferred from one computer to another. But for human beings, once a person leaves his post, no matter how much experience he has, he cannot completely transfer it to another person mind. Third, it has the characteristics of self-organization and self-learning, which can be learned through training. Fourth, the computer has a powerful computing function, and ultimately analyzes the law of disease and various trends. All these eventually lead to the rapid development of computer-aided diagnosis technology. Computer-aided diagnosis technology can help us make more objective and accurate diagnoses and prognoses of lymph node diseases.

### 1.1. The computer-aided diagnosis system of lymph node ultrasonography

The computer-aided diagnosis system of lymph node ultrasonography was developed by Fudan University. The operating environment is a WinXP system, with programming mixed with Visual C++ 6.0 and MATLAB 7.0 (Fig. 1). The system requires that the data of lymph node ultrasound images be in bitmap file format. The AI system was validated through training. Its main functions are ultrasonic image segmentation, quantitative feature extraction, and classification recognition.

**Figure 1.**
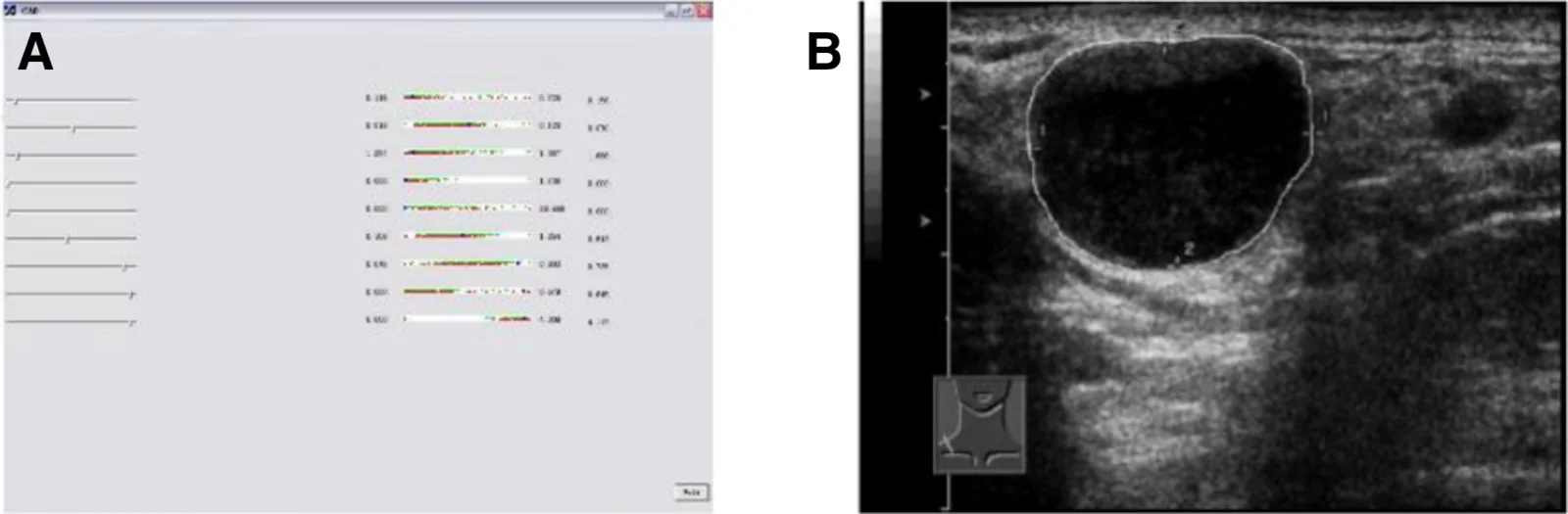
Introduction of computer-aided diagnosis system for lymph node ultrasonography (A) Interface of the system (B) Segmentation of the ultrasonic images.

Ultrasonic image segmentation involves drawing the outline and medulla of lymph nodes with a mouse, saving them separately, and then extracting quantitative features. Ultrasonic image feature extraction refers to the system’s ability to extract 8 kinds of quantitative parameters, including margin, appearance, medulla proportion, medulla distribution, echogenicity, vascular density, and vascular pattern features, and save the corresponding parameters.

After loading the training samples, the system will train the rough interval support vector machine with the read training sample set. The system will display the maximum and minimum values of each feature parameter in the samples, as well as the relative position of each sample in each feature space. After extracting the quantitative features of the analyzed lymph nodes, if the training sample set has been loaded at this time, the system will display the relative positions of the quantitative features of the currently analyzed lymph nodes in the corresponding feature space, and the currently analyzed samples will be displayed with blue dots. Moreover, according to the position of the unknown samples in the feature space relative to the classification plane and the training samples, the system will judge whether it is benign or malignant, and the probability that the unknown samples may be benign or malignant is given.

### 1.2. Characteristic ultrasonic parameters

The computer-aided diagnosis system of lymph node ultrasonography quantifies the following features.

1. External features This diagnostic system uses the Fourier form factor to quantitatively describe the regularity of shape.^[8]^
  The definition is: $FF=1\frac{\sum_{k=-N/2+1}^{N/2\;}\left\|NFD(k)\right\|/\left\|k\right\|}{\sum_{k=-N/2+1}^{N/2\;}\left\|NFD(k)\right\|}$
2. Margin The system uses margin to describe boundary features quantitatively. The definition is: $A=\frac{1}{d_{\max}}\sqrt{\frac{1}{N}\sum_{j=0}^{N-1}\frac{d^{2}(\;j)}{n}}$.
3. Appearance: The ratio of length to diameter was used to quantitatively describe the morphological characteristics. In the same section, the longitudinal diameter (L) of lymph nodes is divided by the transverse diameter (s), i.e., L/ s.
4. Medulla proportion: In the range of lymph node contour, the ratio of medullary pixels to the total number of lymph node pixels is used as a quantitative feature to describe the medulla proportion.
5. Medulla distribution: The distance difference between the two lymph node contour points distributed radially with the medullary centroid and the medullary centroid was calculated. The average distance difference of all such contour point pairs was used as a parameter to describe the medulla distribution characteristics.
6. Echogenicity: Firstly, mathematical morphology was used to expand the contour of lymph nodes to obtain the peripheral range of the original contour, in which the scale of structural elements used for expansion was 1/ 3 of the length of short diameter. The average gray value of the pixels inside the lymph node and the average gray value of the pixels around the lymph node were calculated, and the ratio of the two was used to quantitatively describe the echogenicity characteristics.
7. Echogeneity: The texture feature parameter (Haralick texture feature) calculated from the gray-level co-occurrence moment is used to quantitatively describe the echogeneity in lymph nodes.
8. Vascular density: The ratio of the number of blood flow pixels in the lymph node to the number of pixels in the whole lymph node contour is used to describe the characteristics of vascular density.
9. Vascular pattern: The color blood flow pixels in lymph nodes are projected onto the contour of lymph nodes; that is, the nearest contour point is found for each blood flow point. By calculating the probability of each contour point being projected, the probability distribution histogram of the contour point being projected can be obtained, as shown in Figure 2A–D. The entropy of projection probability distribution was used as a parameter to quantitatively describe the vascular pattern.

**Figure 2.**
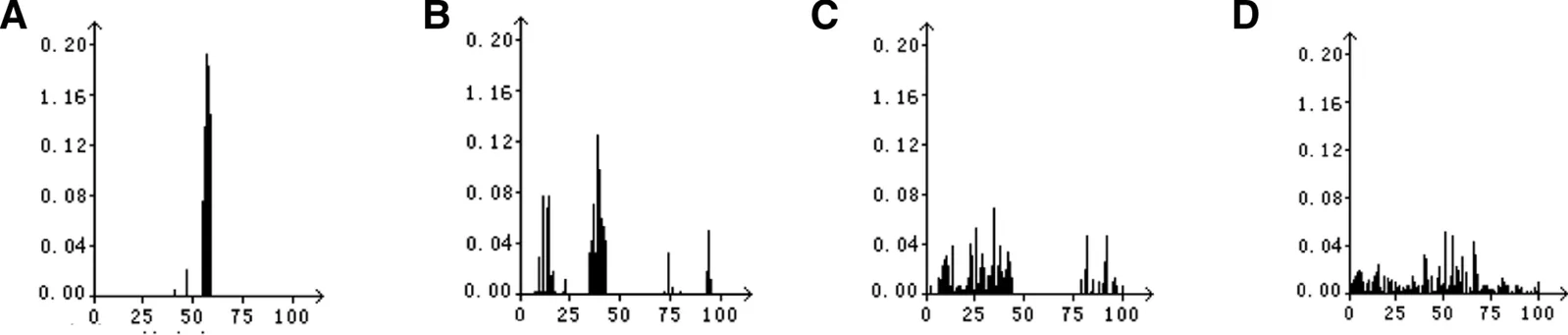
Histogram of probability distribution in projection of contour points of various types of blood flow distribution (A) Histogram of gate type (B) Histogram of peripheral type (C) Central histogram (D) Mixed histogram.

## 2. Materials and methods

### 2.1. Research objects

This study was a retrospective observational study. The study was approved by the Ethics Committee of Shaoxing Second Hospital.

#### 2.1.1. Inclusion criteria

Age ≥18 years old; Cervical lymphatic enlargement (maximum diameter > 8mm in region II, maximum diameter >5mm in other regions); Preoperative ultrasound examination of cervical lymph node was performed, and the image and report data were complete; and The cervical lymph node has a clear postoperative pathological report.

#### 2.1.2. Exclusion criteria

Incomplete ultrasound image data or poor image quality could not provide important information such as the size of the cervical lymph node, edge condition, internal echo, presence of a lymphatic portal, and blood supply type. Pathology could not determine the nature of the cervical lymph node, and the pathological findings of the cervical lymph nodes were inconsistent with those of the lymph nodes indicated by preoperative ultrasound.

From January 2018 to March 2020, 185 patients with lymphadenopathy were studied in our hospital, including 87 males and 98 females. Their ages ranged from 28 to 56 years old, with an average of 45 ± 7 years old. Each case of lymph node lesion was confirmed by puncture or surgical pathology. According to the final pathological results, the lymph nodes were divided into two groups: the benign group and the malignant group. Among them, 110 cases were benign, and 75 cases were malignant (Table 1).

**Table 1:** Patient characteristics.

Variables	No.
Number of patients	185
Gender	
Men (%)	87 (47.0%)
Women (%)	98 (53.0%)
Age (yr, range)	45 ± 7 (28–56)
AFP positive	3 (1.6%)
CEA positive	5 (2.7%)
Interventional treatment	103 (55.7%)
Surgical treatment	82 (44.3%)
Benign pathological results	110 (59.5%)
Malignant pathological results	75 (40.5%)
Max diameter (cm, range)	5.3 (1.1–5.3)
Multiple nodules (>3)	36 (19.5%)
Total diameter >3 cm	17 (9.2%)

### 2.2. Instruments and methods

The linear array probe had a frequency of 5 to14 MHz, with the function of local image magnification. The ultrasonic observation depth was 3 to 6 cm, and the lymph nodes were clearly displayed. The patient took the supine position, fully exposed the tested part, adjusted the instrument to its best state, and scanned the lymph nodes of the corresponding lesions in multiple sections. During the scanning, the probe gently pressed the skin and was coated with a coupling agent. The margin, appearance, medulla proportion, medulla distribution, echogenicity, echogeneity, vascular density, and vascular pattern of the lymph nodes were comprehensively understood by moving the probe parallel in longitudinal, transverse, and oblique sections. The maximum long-axis section was obtained and photographed or stored on a hard disk for measurement and analysis. Then, the blood flow in and around the lymph nodes was observed, and the ultrasound images with the most abundant color blood flow signals were taken or stored on the hard disk for measurement and analysis.

### 2.3. Statistical methods

SPSS 12.0 software was used to test the data. The measured values are expressed as X ± S. A nonparametric rank sum test was used to examine the relationship between the parameters of different pathological lymph nodes. A paired chi-square test was used to compare the rates. There was a significant difference between the two groups (*P* < .05).

## 3. Results

The sensitivity, specificity, and accuracy of the computer-aided diagnosis system were 80.0%, 80.9%, and 80.5%, respectively. The classification and recognition ability of the system for benign and malignant lymph nodes is shown in Table 2. The echogeneity of the lymph node had the best classification and recognition ability for benign and malignant lymph nodes. The appearance also had good classification and recognition ability for benign and malignant lymph nodes. However, the margin of the lymph node contributed less to the classification and recognition ability of benign and malignant lymph nodes.

**Table 2:** Classification and recognition ability of benign and malignant lymph nodes for each quantitative characteristic parameter.

Ultrasonic characteristics	Quantitative characteristic parameters	A_z_ value
Margin*	Margin	0.667
Appearance*	L/ s	0.805
Medulla proportion*	Medulla proportion	0.717
Medulla distribution*	Medulla distribution	0.736
Echogenicit*	Echogenicit	0.718
Echogeneity*	Haralick texture feature	0.823
Vascular density*	Vascular density	0.765
Vascular pattern*	Entropy of projection probability distribution	0.706

* There were significant differences between benign and malignant groups. A_z_ means area under the curve. L/ s means longitudinal diameter divided by the transverse diameter.

## 4. Discussion

The computer-aided diagnosis system for lymph node ultrasonography extracted and analyzed all parameters, and there were significant differences between benign and malignant lymph nodes regarding margin, appearance, medulla proportion, medulla distribution, echogenicity, echogeneity, vascular density, and vascular pattern.

In this diagnosis system, the Fourier form factor (FF) value is used to describe the regularity of the shape. The more the shape is rounded, the smaller the FF value is. Only when there are more “breaks” in the outline line, the FF value will change greatly.^[8]^

The margin of benign lymph nodes was higher than that of malignant lymph nodes. Most of the benign lymph nodes have clear and regular boundaries, a smooth, regular, and clear capsule, and clear boundaries with surrounding tissues.^[9,10]^ The low margin of malignant lymph nodes may be due to the replacement of normal lymph node tissue by tumor cells, the increase of acoustic impedance difference between lymph nodes and surrounding tissue, or adhesion with surrounding tissue, or tumor invasion outside the capsule.^[11,12]^

In this study, most of the benign lymph nodes were spindle-shaped and oval, and the longitudinal diameter divided by the transverse diameter ratio was higher than that of the malignant lymph nodes. The malignant lymph nodes were multicentric nodular infiltration, which led to the morphological changes of lymph nodes. The swelling was spherical, round, or elliptical, or irregular, with low longitudinal diameter divided by the transverse diameter.

In our study, most of the benign cervical lymph nodes maintained their original shape, which was fusiform and oval, and L/S was higher than that of the malignant group. Malignant cervical lymph nodes showed polycentric nodular infiltration, resulting in morphological changes of cervical lymph nodes, which were swollen spherical, round or elliptical, or irregular in shape, and L/S was low. Veverkova *L* reported that the most specific signs of benign and malignant lymph nodes were the nonuniformity of the lymph node cortex, as well as the absence of fatty hilum and perinodal edema.^[13]^Lymph node metastases with lower L/T ratio, peripheral edema, and peripheral vascularization were significantly more frequent. Biopsy of these lymph nodes is necessary to confirm or rule out metastasis, especially if it affects the type of treatment. Loonis AT reported that multivariate analysis showed that benign and malignant lymph nodes were significantly correlated with portal replacement and cortical thickness >3 mm in ultrasonic images (*P* < .05). These imaging criteria, together with the higher imaging diagnosis (grade II and III) and the breast tumor quadrant, form a Nomogram for calculating the probability of lymph node malignancy.^[14]^ Zheng B reported that there was a significant difference in the ratio of cortical and medullary lymph nodes between the metastatic and non-metastatic groups.^[15,16]^ Yan Shan reported that lymph node L/S was 1.5, and the sensitivity and specificity of ultrasound in differentiating benign and pathological lymph nodes were 71% and 65%. When lymph node L/S was 2, sensitivity increased to 85%, and specificity increased to 86%.^[17]^

The medulla proportion in the benign group was higher than that in the malignant lymph node group. The number of medulla can indicate whether lymph nodes are benign or malignant. Medullary hyper echogenicity of lymph nodes is often a manifestation of benign lymph nodes.^[18]^ The medulla of benign enlarged lymph nodes is larger than that of malignant lymph nodes, and most of them are widened and enhanced. If the cortex is thickened, it is mainly centripetal thickening. The cortical infiltration and thickening of malignant lymph nodes may also invade the medulla, resulting in the disappearance of the medulla.^[19]^

The distance difference between the two lymph node contour points with medullary centroid radial distribution and the medullary centroid is used as a parameter to describe the medullary distribution. When the medulla shifted and the cortex widened unevenly, the distance difference would be larger. However, in the clinic, the normal lymph node medulla enters from the hilum of the lymph node. In the gray-scale image of a lymph node that knocks down the hilum of a lymph node, the system identifies and misjudges it as eccentric displacement of the lymph node medulla.

In this system, texture feature parameters calculated by gray-level co-occurrence moment are used to describe echo uniformity. The pathological features of benign lymph nodes were that the cortex and medulla increased evenly, and most of them showed moderate echo. Malignant lymph nodes infiltrated lymph nodes, and the internal echo was variable and uneven. The whole lymph nodes of lymphoma showed hypoechoic, hypoechoic, or cyst-like type. The metastatic lymph nodes had no normal structure, only hypoechoic, and the medulla disappeared. The echo of metastatic cancer was uneven. Sometimes, there were fine dot hyperechoic areas caused by micro calcification or anechoic areas caused by liquefaction and necrosis.

The ratio of the number of blood flow pixels in the lymph node to the number of pixels in the whole lymph node contour is used to describe the characteristics of blood flow density. Color Doppler flow imaging blood flow density in lymph nodes was divided into 4 grades: 0 to 3.^[20]^ In this paper, the quantitative value of Color Doppler flow imaging in lymph nodes is continuous digital data, which is quantitative analysis, more objective than human-centered analysis, and more sensitive and valuable than grade data in statistical analysis.

According to the findings of color Doppler ultrasound,^[20]^ the vascular patterns of diseased cervical lymph nodes were divided into four types: lymphatic hilar type, central type, marginal type, and mixed type. The main blood vessel pattern in benign cervical lymph nodes is the lymphatic portal type. The blood flow in benign cervical lymph nodes is regular and evenly distributed, mostly in the shape of water grass and regular branches, while malignant cervical lymph nodes show various types. Benign cervical lymph nodes exhibit a single, regular, short rod-like blood flow signal with few branches, which regularly runs along the hilum or medullary part of cervical lymph nodes. In contrast, malignant cervical lymph node tumor cells secrete angiogenic factors to induce distorted trophic vessels, and the internal blood supply is diversified and abundant.^[21]^ The blood flow signal of benign cervical lymph nodes is not as abundant as that of malignant cervical lymph nodes, which may be related to the induction of nutrient vessels inside the tumor, and is consistent with the pathological changes of malignant cervical lymph nodes. Malignant cervical lymph nodes show blood flow signals of multiple nourishing vessels due to tumor growth and the formation of new vessels.^[22,23]^ Malignant cervical lymph nodes often have a diffuse mass of blood vessels with high velocity and uneven distribution. Aladag Kurt S reports that through a single factor analysis, lymph node metastasis was significantly associated with tighter asymmetric cortical thickening (*P* < .0001), cortex to hilum ratio (*p* = .001), cortical thickness (*P* = .001), portal status (*P* < .005), and vascular morphology (*P* < .005). The L/T ratio was statistically significant only in predicting metastasis of diffuse cortical enlarged lymph nodes, and aggregation was observed only in this group (*P* < .05). Multivariate analysis showed that lymph node metastasis was significantly associated with asymmetric cortical thickening (*P* = .001), cortex to hilum ratio (*P* = .005), and vascular morphology (*P* < .0001). Asymmetrical enlargement of the proximal mammary cortex, C/H ratio, and abnormal microvascular morphology are independent predictors of lymph node involvement.^[24]^ Metastatic cervical lymph nodes often present subcapsular blood flow signals, that is, peripheral blood flow signals. Cervical lymph nodes with lymphoma show mixed blood flow signals. This is caused by the different infiltration modes of tumor cells in the cervical lymph nodes.^[25]^

In this paper, the entropy of projection probability distribution is used to describe vascular distribution. The higher the disorder of the elements in a system, the higher the entropy. The entropy of projection probability distribution in malignant lymph nodes is higher than that in benign lymph nodes. Metastatic lymph node tumor cells invade lymph nodes from the outside, form tumor nests under the capsule, and then slowly infiltrate into the whole lymph node. Lymphoma lymph nodes start from the inside of lymph nodes. Depending on the invasiveness of the tumor, they may never reach the capsule, or they may infiltrate to the periphery, accumulating in the whole lymph node. Therefore, blood flow around metastatic lymph nodes is more common, especially the nutrient vessels passing through the capsule have high specificity for metastatic lymph nodes, which is helpful for clinical diagnosis. Some malignant lymph nodes have no blood flow signal.

The application of artificial intelligence in the medical field requires a large amount of data, but many data sources are unreliable and of varying quality, which can have a negative impact on the accuracy of machine learning algorithms. Furthermore, there are high security and privacy protection requirements for data in the medical field. If artificial intelligence systems cannot meet these requirements, it may lead to data leakage, affecting the privacy and security of patients. Moreover, data in the medical field has complex causal relationships, and most machine learning algorithms can only handle statistical relationships, which can affect the accuracy of artificial intelligence systems.

In a word, the artificial intelligence computer-aided diagnosis system of superficial lymph node ultrasound images developed by Fudan University has definite value for the parameter extraction and benign and malignant classification of superficial lymph node ultrasonography. According to superficial lymph node ultrasonography, the characteristic parameters of benign and malignant lesions can be selected, which can meet the requirements of clinical application. The limitations of this study are insufficient sample size and the inevitable errors and subjectivity caused by manual segmentation methods. More prospective studies are needed in the future to confirm the clinical feasibility of the system. Further research to increase sample size to improve system accuracy is also needed.

## Author contributions

**Conceptualization:** Tingting Jin, Liying Wang.

**Methodology:** Liying Wang.

**Supervision:** Shusen Zheng.

**Writing – original draft:** Liying Wang.

**Writing – review & editing:** Liying Wang, Shusen Zheng.
